# Community-Dwelling Older Adults’ Readiness for Adopting Digital Health Technologies: Cross-Sectional Survey Study

**DOI:** 10.2196/54120

**Published:** 2024-04-30

**Authors:** Dietmar Ausserhofer, Giuliano Piccoliori, Adolf Engl, Angelika Mahlknecht, Barbara Plagg, Verena Barbieri, Nicoletta Colletti, Stefano Lombardo, Timon Gärtner, Waltraud Tappeiner, Heike Wieser, Christian Josef Wiedermann

**Affiliations:** 1 Institute of General Medicine and Public Health Bolzano Italy; 2 Claudiana Research College of Healthcare Professions Claudiana Bolzano Italy; 3 Provincial Institute of Statistics (ASTAT) Bolzano Italy

**Keywords:** frail older adults, Italy, Italian, Europe, European, digital health, health technologies, health technology, telemedicine, telehealth, eHealth, e-health, adoption, readiness, usage, survey, surveys, questionnaire, questionnaires, robotics, readiness, adoption, cross-sectional study, population-based survey, stratified probabilistic sampling, gerontology, geriatric, geriatrics, older adult, older adults, elder, elderly, older person, older people, ageing, aging

## Abstract

**Background:**

Digital health technologies offer the potential to improve the daily lives of older adults, maintain their health efficiently, and allow aging in place. Despite increasing evidence of benefits and advantages, readiness for adopting digital interventions among older people remains underexplored.

**Objective:**

This study aims to explore the relationships between sociodemographic-, health-, and lifestyle-related factors and technology use in everyday life and community-dwelling older adults’ readiness to adopt telemedicine, smartphones with texting apps, wearables, and robotics.

**Methods:**

This was a cross-sectional, population-based survey study with a stratified probabilistic sample of adults aged 75 years or older living in South Tyrol (autonomous province of Bolzano/Bozen, Italy). A random sample of 3600 community-dwelling older adults living at home was invited to complete a questionnaire including single items (older adults’ readiness to use health technology) and scales (PRISMA-7; Program of Research on Integration of Services for the Maintenance of Autonomy). Descriptive and logistic regression analyses were performed to analyze the data.

**Results:**

In total, 1695 community-dwelling older adults completed the survey (for a response rate of 47%). In terms of potential digital health technology adoption, wearable devices were favored by 33.7% (n=571), telemedicine by 30.1% (n=510), smartphones and texting apps by 24.5% (n=416), and assistant robots by 13.7% (n=232). Sociodemographic-, health- and lifestyle-related factors, as well as the use of technology in everyday life, played a significant role in explaining readiness to adopt digital health technologies. For telemedicine, age ≥85 years (odds ratio [OR] 0.74, 95% CI 0.56-0.96), financial constraints (OR 0.68, 95% CI 0.49-0.95), and less than 2 hours of physical activity per week (OR 0.75, 95% CI 0.58-0.98) were associated with nonreadiness, while Italian-speaking participants (OR 1.54, 95% CI 1.16-2.05) and those regularly using computers (OR 1.74, 95% CI 1.16-2.60), smartphones (OR 1.69, 95% CI 1.22-2.35), and the internet (OR 2.26, 95% CI 1.47-3.49) reported readiness for adoption.

**Conclusions:**

Community-dwelling older adults display varied readiness toward the adoption of digital health technologies, influenced by age, mother tongue, living situation, financial resources, physical activity, and current use of technology. The findings underscore the need for tailored interventions and educational programs to boost digital health technology adoption among community-dwelling older adults.

## Introduction

As global populations age, health care systems worldwide grapple with the challenge of providing personalized, efficient, and integrated care for an increasing proportion of older adults, with the ultimate goal of facilitating “healthy ageing-in-place” [1]. Digital health technologies offer potential advancements to improve various older adults’ health outcomes and access to health care services [2]. There is increasing evidence of the benefits of digital health interventions for community-dwelling older adults in terms of physical function, cognitive performance, depression, behavioral and psychological symptoms of dementia, and overall quality of life [3-6]. Digital health technologies, such as smartphone-based mobile apps or wearables, allow the measurement and tracking of clinical parameters, such as pain, fatigue, fever, arrhythmias, slower walking speed, and insufficient physical activity to prevent or improve the management of chronic diseases, frailty, morbidity, and mortality [7]. For instance, in older adults with type 2 diabetes mellitus, digital health interventions (ie, mHealth) have been shown to be effective in improving cardiometabolic outcomes [8].

Despite technological advancements and the benefits of digital interventions, the adoption of digital health technologies remains low and inconsistent among older persons, suggesting that underlying factors influence their acceptance, adoption, and use [9]. For example, older adults’ adoption of new digital health technologies, such as social robots to combat social isolation and loneliness [10], depends on multiple factors related to technology, psychological, social and personal aspects, costs, and the environment [11]. Personal factors, including life satisfaction, social relationships, self-perception of health, and everyday activities, are integral facets of an individual’s life, especially in their senior years. These aspects not only shape their overall well-being but also their openness to embracing and readiness to adopt and use potentially beneficial digital health technologies [12]. As the intersections of these domains with digital health adoption have been underexplored, particular attention needs to be given to studying the antecedents of digital health technology adoption, including older adults’ readiness [11]. In Italy, a potential digital health gap among older adults due to infrastructural issues and the lack of digital skills have been described, with differences between age groups and educational levels [13]. In a cross-sectional survey study, less than half of the 1002 respondents were aware of telemedicine services in their region [14]. According to health care professionals, some groups of patients experience difficulties in accessing and using digital health technologies due to sociocultural factors, technological and linguistic challenges, and the absence of caregivers [15]. Yet, little is known about the adoption and use of digital health technologies by community-dwelling older adults. In this study, we aimed to explore the relationships between sociodemographics, health- and lifestyle-related factors, and technology use in their everyday life and community-dwelling older adults’ readiness to adopt digital health technologies, that is, telemedicine, smartphones with texting apps, wearables, and robotics. A deeper understanding of these intersections can inform the design, implementation, and evaluation of digital health technology interventions that resonate closely with the needs and preferences of older adults. Such insights can catalyze the development of more target group-oriented digital health strategies, ensuring not only better health outcomes but also improved quality of life for community-dwelling older adults in the digital age.

## Methods

### Study Design

This cross-sectional, population-based survey study was conducted jointly by the Provincial Institute of Statistics-ASTAT (Istituto Provinciale di Statistica – Landesinstitut für Statistik) and the Institute of General Medicine and Public Health in the Autonomous Province of Bolzano, South Tyrol between March 1 and May 30, 2023.

### Setting and Sample

South Tyrol, the autonomous province of Bolzano, is part of the Trentino–Alto Adige region in Italy, next to Austria (total population: 534,912), with approximately 70% German-speaking, 25% Italian-speaking, and 5% other languages. The target population of the survey comprised approximately 51,000 individuals residing in South Tyrol aged 75 years and older. A stratified probabilistic sampling method was used in this study. The ASTAT randomly selected 3600 community-dwelling adults aged ≥75 years, stratified by age (75-84 years, 85 years and older), sex (male and female), and residency (municipalities), from the register of the current resident population in the whole province. To ensure an adequate level of precision, the sampling of the 3600 individuals considered the distribution of and variation between the strata.

Excluded from the survey were individuals permanently residing in senior living facilities and those who, due to health reasons, were unable to complete the questionnaire independently or with the aid of a family member.

### Participant Survey

The participant survey was designed collaboratively by the ASTAT and the Institute of General Medicine and Public Health, based on a similar survey study conducted in 2013 [16] and the INSPIRE population survey applied in Switzerland [17]. The German and Italian language versions, translated from the ASTAT, were reviewed for language equity by the research group at the Institute for General Medicine and Public Health. The final versions were checked and approved by the local health and social authorities. As the survey questionnaire included instruments and items previously used (in 2013 edition of the survey and INSPIRE population survey), we conducted pretesting with 10 older adults aged 75 years and older. These participants were invited to complete the questionnaire and to provide oral feedback regarding the clarity of questions, difficulty of completion, and the technical functionality of the web-based survey to the research group by phone.

Older adults’ readiness for adoption of digital health technologies was assessed using single items, asking to rate on a 5-point Likert scale (yes/maybe/no/I do not understand what this means/I am already using it) the following four technologies: (1) telemedicine, that is, communicating with your doctor via video or smartphone; (2) smartphone with text messaging apps, that is, reminding you of your clinical condition or taking medications, providing information on how to manage your condition; (3) wearable devices, that is, monitoring your heart rate, blood glucose, physical activity, or SOS device; and (4) assistive robots, that is, supporting you at home for taking medications, or recognizing emergencies.

Participants’ sociodemographics included age (birth year), sex (male/female), native tongue (German/Italian/Ladin/Others), citizenship (Italy/other country), educational level (Below Highschool/Highschool or higher), community and region of origin (rural/urban), living situation (alone/with spouse or family member), children (yes/no), financial resources (excellent or good/adequate/insufficient or low), and overall optimism (yes/no).

Health- and lifestyle-related factors included self-reported health status (poor or moderate/good or very good), frailty (no=0-3 points/yes=4-7 points on PRISMA-7 [Program of Research on Integration of Services for the Maintenance of Autonomy], [18]), physical activity (2 hours or more a week/ less than 2 hours a week/ never), use of home care assistance (eg, from family, nursing team, or private family assistant; yes/no).

Technology use in everyday life was assessed by asking participants if they had already used computers, tablets (yes/no), smartphones (yes/no), or the internet (yes/no).

### Data Collection

Letters were mailed from the ASTAT to the randomly sampled participants to inform them about the study and to invite them to voluntarily participate by completing the survey alone or with the aid of a family member. Completion of the survey was possible by one of the following: (1) web-based self-completion, (2) paper-based self-completion, or (3) telephonic interviews with collaborators from the ASTAT. One month after the first letter, a second letter was sent to inform them about the study and invite them to participate. The web-based survey was created using LimeSurvey [19].

### Ethical Considerations

Ethical approval was obtained from the institutional board of the Institute of General Practice and Public Health, Bolzano, Italy (reference number: 03/2023). All study procedures were in accordance with the 1964 Helsinki Declaration and its amendments, European Union General Data Protection Regulation (679/2016), and Italian Data Protection Law (196/2003). Before filling out the online questionnaire, participants were explicitly asked to provide informed consent. Filling out the paper questionnaire and sending it back by post was considered as participants’ informed consent. Participation was voluntary. All data of the study participants were anonymized to protect their identities.

### Statistical Analysis

Only fully completed questionnaires were included in the statistical analysis, resulting in the exclusion of 73 partially filled-out questionnaires. Descriptive statistics (eg, frequency) were calculated to describe the measured variables. To explore differences between the characteristics of older adults and their readiness to adopt digital health technologies (yes and maybe vs no), we used Fisher exact test. Four binary logistic regression models were used to explore the association between each digital health technology, that is, telemedicine, smartphone and texting apps, wearables and robotics (dependent variables), sociodemographics, health- and lifestyle-related factors, and use of technology in everyday life (independent variable). All analyses were performed using R (version 4.3.1; R Foundation for Statistical Computing) and RStudio (version 2023.6.2.561) with the packages *tidyr* [20] and *lme4* [21]. A *P* value of less than .05 was considered significant.

## Results

### Sample Characteristics

In total, 1695 community-dwelling older adults completed the survey, reflecting a response rate of 47%. As described in Table 1, the majority of participants were female (880/1695, 51.9%), aged between 75 and 84 years (1005/1695, 59.3%), living with partners or family (1179/1695, 69.6%), had at least 1 child (79.3%), and had an educational level below high school (1319/1695, 77.8%). Health status was reported in more than half as poor or moderate (1012/1695, 59.7%), not frail (999/1695, 58.9%), yet receiving home care assistance from family, nursing team, or a private family assistant (1121/1695, 66.1%). Approximately 1 in 3 older adults mentioned using a computer or tablet (524/1695, 30.9%), smartphone (678/1695, 40%), and the internet (656/1695, 38.7%) in their everyday lives.

**Table 1:** Characteristics of study participants (n=1695).

Characteristics		Values, n (%)
**Sex**		
	Female	880 (51.9)
	Male	815 (48.1)
**Age (years)**		
	75-84	1005 (59.3)
	≥85	690 (40.7)
**Native tongue**		
	German	902 (53.2)
	Italian	713 (42.1)
	Ladin	67 (4)
	Other	13 (0.8)
**Citizenship**		
	Italian	1673 (98.7)
	Other	22 (1.3)
**Community**		
	Rural	773 (45.6)
	Urban	992 (54.4)
**Living situation**		
	Living alone	516 (30.4)
	Living with partner or family	1179 (69.6)
**Children**		
	Yes	1344 (79.3)
	No	351 (20.7)
**Educational level**		
	Below highschool	1319 (77.8)
	Highschool or higher	376 (22.2)
**Financial resources**		
	Excellent or good	483 (28.5)
	Adequate	837 (49.4)
	Insufficient or low	375 (22.1)
**Overall optimism**		
	Yes	1433 (84.5)
	No	262 (16.5)
**Health status**		
	Poor or moderate	1012 (59.7)
	Good or very good	683 (40.3)
**Frailty (PRISMA-7)^a^**		
	Yes	574 (33.9)
	No	1121 (66.1)
**Physical activity**		
	2 hours or more a week	733 (43.2)
	Less than 2 hours a week	679 (40.1)
	Never	283 (16.7)
**Home care assistance (eg, from family, nursing team, or private family assistant)**		
	Yes	999 (58.9)
	No	696 (41.1)
**Using computer or tablet**		
	Yes	524 (30.9)
	No	1171 (69.1)
**Using smartphone**		
	Yes	678 (40)
	No	1017 (60)
**Using internet**		
	Yes	656 (38.7)
	No	1039 (61.3)

^a^PRISMA-7: Program of Research on Integration of Services for the Maintenance of Autonomy.

### Older Adults’ Readiness for Digital Health Technologies Adoption

Table 2 describes the readiness of older adults to adopt health technology. Overall, readiness to adopt technological solutions in health care among older people varies significantly based on technology type. In terms of current technology use, telemedicine emerged as the most common digital health technology used by 5.4% (n=91) of the participants. In terms of potential adoption, wearable devices were favored by 33.7% (n=571), telemedicine by 30.1% (n=510), smartphones and texting apps by 24.5% (n=416), and assistant robots by 13.7% (n=232). At the other end of the adoption spectrum, there was clear reluctance, with 44.7% (n=757) against smartphone and text apps, 39.5% (n=669) against telemedicine, and 52.4% (n=888) against the use of robotics.

**Table 2:** Older adults’ readiness for health technology adoption (N=1695).

Characteristics	Yes	Maybe	No	I do not understand what this means	Already using it
Telemedicine, n (%)	510 (30.1)	342 (20.2)	669 (39.5)	83 (4.9)	91 (5.4)
Smartphone and texting apps, n (%)	416 (24.5)	393 (23.2)	757 (44.7)	87 (5.1)	42 (2.5)
Wearable devices, n (%)	571 (33.7)	541 (31.9)	489 (28.8)	60 (3.5)	34 (2)
Assistant robots, n (%)	232 (13.7)	459 (27.1)	888 (52.4)	108 (6.4)	8 (0.5)

### Group Differences in Older Adults’ Readiness for Adopting Digital Health Technologies

The tables in Multimedia Appendix 1 present the sociodemographics of the participants and highlight group differences between those who are ready to adopt digital health technology (either responded “Yes” or “Maybe”) and those who responded “No”. In summary, age, educational level, living situation, financial resources, physical activity, and technology use in everyday life significantly influenced older adults’ readiness to integrate various digital health technologies into their lives.

Age plays a key role in digital health technology adoption readiness. In particular, 72.4% (n=697) of older adults between the ages of 75 and 84 years showed readiness to adopt wearables. Living arrangements also had an impact on digital health technology adoption, with individuals living with a partner or family showing higher readiness to integrate all forms of digital health technologies than their counterparts living alone. Similarly, older adults with an educational level of high school or higher; those reporting having adequate financial resources; being physically active; and older adults already using computers, tablets, smartphones, and the internet reported higher readiness to adopt all 4 digital health technologies. In terms of gender differences, except for robotics, more than half of the men reported being ready to adopt digital health technologies and higher readiness than women to use telemedicine and smartphones. Italian-speaking adults living in urban areas who were more optimistic about the future, with good or very good health status, and were not frail, were more likely to adopt 2 out of 4 digital health technologies, namely telemedicine and smartphones with texting apps. Regarding frailty as assessed by PRISMA-7, we observed that nonfrail individuals reported higher readiness to adopt telemedicine, as well as smartphone and texting apps compared with frail individuals.

### Factors Influencing Older Adults’ Readiness for Adopting Digital Health Technologies

Tables 3 and 4 present the results of multiple logistic regression analyses examining the association between older people’s readiness to use 4 digital health technologies (telemedicine, smartphone with texting app, wearables, and assistant robot) and various sociodemographic, health- and lifestyle-related factors, and use of technology in everyday life.

**Table 3:** Multiple logistic regression analyses between older people’s readiness to use telemedicine, smartphones with texting apps and sociodemographics, health- and lifestyle-related factors, and use of technology in everyday life (n=1521).

Characteristics				Telemedicine					Smartphone with texting app		
				OR^a^ (95% CI)		*P* value		OR (95% CI)			*P* value
**Sociodemographic factors**											
	Female (reference group: male)		1.11 (0.87-1.43)		.40		1.00 (0.78-1.28)			.90	
	Age ≥85 years (reference group: age 75-84 years)		0.74 (0.56-0.96)		.03		0.75 (0.57-0.98)			.03	
	**Native tongue (reference group: German)**										
		Italian	1.54 (1.16-2.05)		.003		1.41 (1.07-1.87)			.02	
		Ladin and others	1.55 (0.90-2.69)		.11		1.16 (0.67-2.00)			.60	
	Rural community (reference group: urban community)		1.03 (0.78-1.36)		.80		0.99 (0.76-1.30)			.90	
	Living alone (reference group: living with family)		0.95 (0.74-1.23)		.70		0.95 (0.74-1.23)			.70	
	Educational level (reference group: below high school)		1.18 (0.86-1.63)		.30		1.06 (0.78-1.45)			.70	
	**Financial resources (reference group: excellent or good)**										
		Adequate	0.84 (0.64-1.10)		.20		0.95 (0.73-1.25)			.70	
		Insufficient or low	0.68 (0.49-0.95)		.02		0.83 (0.60-1.16)			.30	
**Health-related factors**											
	Overall optimism (reference: no)		1.22 (0.88-1.68)		.20		1.21 (0.88-1.66)			.30	
	Good or very good health status (reference: poor or moderate)		0.98 (0.75-1.28)		.90		0.96 (0.74-1.24)			.70	
	No frailty (reference: frailty)		0.82 (0.59-1.15)		.30		0.88 (0.63-1.23)			.50	
	**Physical activity (reference group: 2 hours or more a week)**										
		Less than 2 hours a week	0.75 (0.58-0.98)		.034		0.81 (0.63-1.05)			.12	
		Never	0.72 (0.50-1.03)		.071		0.59 (0.41-0.85)			.004	
	Home care assistance (reference group: no assistance)		0.77 (0.58-1.02)		.065		0.90 (0.68-1.18)			.40	
**Technology-related factors**											
	Use of computer, tablet (reference: no use)		1.74 (1.16-2.60)		.008		1.55 (1.05-2.28)			.03	
	Use of smartphone (reference: no use)		1.69 (1.22-2.35)		.002		1.68 (1.23-2.29)			.001	
	Use of internet (reference: no use)		2.26 (1.47-3.49)		<.001		2.66 (1.77-4.03)			<.001	

^a^OR: odds ratio.

**Table 4:** Multiple logistic regression analyses between older people’s readiness to use wearables, assistant robots and sociodemographics, health- and lifestyle-related factors, and use of technology in everyday life (n=1521).

Characteristics			Wearables		Assistant robot	
			OR^a^ (95% CI)	*P* value	OR (95% CI)	*P* value
**Sociodemographic factors**						
	Female sex (reference group: male)		1.25 (0.98-1.60)	.07	1.02 (0.82-1.28)	.80
	Age ≥85 years (reference group: 75-84 years)		0.78 (0.59-1.01)	.06	0.71 (0.55-0.92)	.009
	**Native tongue (reference group: German)**					
		Italian	1.05 (0.80-1.39)	.70	0.93 (0.72-1.20)	.60
		Ladin and others	0.99 (0.59-1.68)	.90	1.40 (0.86-2.28)	.20
	Rural community (reference group: urban community)		1.03 (0.79-1.35)	.80	1.05 (0.82-1.35)	.70
	Living alone (reference group: living with family)		0.75 (0.59-0.96)	.02	0.84 (0.66-1.06)	.15
	Educational level (reference group: below high school)		1.08 (0.79-1.48)	.60	0.95 (0.72-1.26)	.70
	**Financial resources (reference group: excellent or good)**					
		Adequate	0.99 (0.76-1.30)	.90	0.84 (0.65-1.07)	.20
		Insufficient or low	0.76 (0.55-1.04)	.09	0.85 (0.62-1.15)	.30
**Health-related factors**						
	Overall optimism (reference: no)		0.98 (0.71-1.34)	.90	1.08 (0.80-1.45)	.60
	Good or very good health status (reference: poor or moderate)		0.88 (0.68-1.14)	.30	1.01 (0.79-1.27)	.90
	No frailty (reference: frailty)		0.79 (0.57-1.10)	.20	0.94 (0.69-1.28)	.70
	**Physical activity (reference group: 2 hours or more a week)**					
		Less than 2 hours a week	0.79 (0.61-1.02)	.08	0.87 (0.69-1.11)	.30
		Never	0.46 (0.33-0.65)	<.001	0.76 (0.54-1.06)	.11
Home care assistance (reference group: no assistance)			0.64 (0.48-0.85)	.002	0.63 (0.48-0.81)	<.001
**Technology-related factors**						
	Use of computer, tablet (reference: no use)		1.27 (0.85-1.88)	.20	1.18 (0.84-1.68)	.30
	Use of smartphone (reference: no use)		1.36 (0.98-1.89)	.07	1.31 (0.97-1.78)	.08
	Use of internet (reference: no use)		1.19 (0.77-1.83)	.40	1.30 (0.88-1.93)	.20

^a^OR: odds ratio.

As reported in Table 3, higher age and insufficient financial resources were associated with lower readiness to adopt telemedicine, as well as being active for less than 2 hours a week. Italian-speaking older adults and everyday users of computers or tablets, smartphones, and internet reported significantly higher readiness to adopt telemedicine.

For smartphones with texting apps, adults aged ≥85 years with no physical activity at all were reported to be less likely to adopt this technology, while users of computers, tablets, smartphones, and the internet reported higher readiness (see Table 4).

Living alone, never performing physical activity, and being in need of home care assistance were all associated with lower readiness to adopt wearables. Regarding assistant robots, older adults aged aged ≥85 years and those already receiving home care assistance were less likely to adopt this digital health technology.

## Discussion

### Principal Findings

The integration of digital health technologies in health care has gained increasing prominence in recent years to improve access to and delivery of services, especially among older adults, who often face unique health care needs. With this cross-sectional, population-based survey study, we aimed to investigate the readiness of community-dwelling older adults in South Tyrol, Italy, to adopt 4 types of digital health technologies. Our study revealed that more than half of the older adults reported readiness to adopt telemedicine and wearable devices. However, a large portion responded with nonreadiness to adopt digital health technologies, that is, smartphones with texting apps or assistant robots. Sociodemographic, health- and lifestyle-related factors, and the use of technology in everyday life played a significant role in explaining older adults’ readiness to adopt digital health technologies.

Our finding on community-dwelling older adults’ readiness to adopt digital health technologies in South Tyrol aligns with a growing body of literature that highlights the increasing acceptance and willingness of older adults to embrace digital health care solutions [22]. In this context, it is evident that readiness to adopt digital health technologies, as observed in South Tyrol’s older population, is part of a more extensive global shift toward digital health technology use. This shift in attitude may be attributed to various factors, including the greater accessibility and user-friendliness of digital devices and health care apps as well as the increasing emphasis on and necessity to use technological solutions during the COVID-19 pandemic [23]. As the use of technology has increasingly become a crucial element of everyday life, older adults seem to embrace the potential benefits of digital health technologies to meet their health care needs, such as improved access to services, enhanced convenience, and better management of chronic conditions, thus playing a crucial role in fostering healthy aging, enhancing social inclusion, and facilitating independent living [24].

Our study revealed a higher adoption readiness for wearables and telemedicine compared with smartphones, texting apps, and assistant robots. One possible explanation for this is the perceived direct health benefits and simplicity associated with wearables and telemedicine. Telemedicine directly addresses the challenges of physical mobility, frequent clinic visits, and waiting times, thereby offering a convenient alternative [23]. Wearables, often designed with user-friendly interfaces, provide real-time health monitoring and a sense of security to older people. In contrast, texting apps may seem less intuitive to a generation that is less accustomed to digital communication, with the added layer of concerns about miscommunication or misunderstanding medical advice. Moreover, older adults are cautious about sharing health-related information online because of privacy and security concerns [25]. Assistant robots, being relatively newer innovations, might invoke apprehensions regarding complexity, safety, and potential dependency. While older adults acknowledge the future potential, readiness to adopt assistant robots is still low because of perceived barriers, such as mismatch between needs and solutions offered by the robots, usability factors, and lack of experience with technology [26].

Our results demonstrate that sociodemographics, health- and lifestyle-related factors, and the use of technology in everyday life play a significant role in shaping older adults’ readiness to adopt digital health technologies. While several sociodemographic, health, and lifestyle-related factors showed significant relationships in simple regression models, their associations were often attenuated or eliminated in multiple regression models. For instance, lower age (below 85 years) was the most consistent sociodemographic factor explaining higher readiness to adopt 3 out of 4 digital health technologies, while higher education or financial resources played a less important role. In our study, Italian-speaking older adults were more inclined to adopt telemedicine, which might be related to higher awareness or potentially more available Italian-language information and campaigns, for example, in television, promoting digital health technology. As language mirrors cultural, societal, and regional differences within South Tyrol, it may play an important role in the acceptance and use of digital health technologies. Recognizing the influence of linguistic and cultural differences on digital health technology adoption can guide future interventions by emphasizing multilingual support and culturally tailored educational and outreach programs.

The association between technology use in everyday life (ie, computer/tablet, smartphone, and internet) and older adults’ readiness to adopt digital health technologies emphasizes the importance of technology literacy, that is, the ability to use, comprehend, manage, and analyze technology safely, effectively, and responsibly. The pronounced readiness among “tech users” in the older age underscores the importance of technological familiarity. Recent research from China has revealed that overcoming technology anxiety is essential in enhancing the adoption and continued use of wearable health technologies among older adults [27]. While developing credible digital health apps that require minimal internet navigation skills, patient education, and collaborative efforts to address access and affordability are urgently warranted [28], tailored training sessions or workshops on basic technology use could substantially bridge the readiness gap. Similarly, health care professionals need to be prepared for the adoption and use of digital health technologies. A recent survey among clinical and nonclinical staff in general practice in South England revealed a significant difference between the self-reported competence in using digital health technologies, with the lowest readiness for using clinical apps and wearables [29].

### Future Implications

Collaboration between all stakeholders, that is, health care providers, technology developers, policymakers, and the older population, is essential to promote the successful integration of digital health technologies into the health care system for older adults in South Tyrol and beyond. Policymakers and health care providers should consider disparities when designing and implementing digital health interventions, and ensure that technologies, including telemedicine, are accessible and affordable to all older adults to avoid health inequities with inequality in the distribution of health care resources among different populations [30]. Moreover, the use of sensors and wearables for remote monitoring as a source of information for chronic disease management needs to be integrated into primary health care processes so that information with clinical value is not lost along the way and by ensuring data protection [31]. Design adaptations and well-thought-out blended alternatives (ie, combining telemedicine with face-to-face support) may be potential solutions to improve older adults’ user engagement with digital health technologies [32].

While our study aimed to describe and explore more in-depth the experiences of individuals aged 75 years and above, future research should focus on evaluating readiness, actual adoption, and use of digital health technologies among older adults of different ages (eg, 55 years and above) over time. Qualitative research is needed to gain an in-depth understanding of the potential facilitators and barriers to adopting and using digital health technologies, which can guide health care providers, technology developers, and policymakers in creating more targeted and user-friendly digital health technology solutions for older adults, ensuring higher adoption rates and beneficial health outcomes. Education and awareness campaigns should address the concerns of older adults, such as safeguards in place to protect their data. Addressing these concerns is crucial for building trust in digital health technologies.

### Limitations

This study has some limitations that need to be considered. First, its cross-sectional design limits our ability to establish causality or track changes in readiness over time. Longitudinal studies are needed to better understand how attitudes toward digital health technologies evolve among older adults. Additionally, the survey was conducted in South Tyrol, Italy, which may limit the generalizability of our findings to other regions or countries with different health care systems and sociocultural contexts. The participants primarily came from a specific age group, predominantly aged 75-84 years. This narrow age range does not offer a comprehensive picture of the general population’s attitudes across a broader age spectrum. We excluded older adults living in residential care facilities as they are more care-dependent and receive 24/7 institutional care, which might limit their readiness, ability, and need to use digital health technologies. Although the response rate (47%) can be considered excellent, this implies that over half of the potential participants did not respond. There could be systematic reasons for nonresponses, which might have introduced bias. Although we aimed to capture a wide range of community-dwelling older adults, we acknowledge that the findings may not be fully representative, as older adults less inclined or unable to embrace technology might not have participated in this study. Finally, the results are quantitative, which, while providing clear metrics, might not explore deeper reasons or barriers behind older adults’ hesitations or willingness to adopt digital health technologies.

### Conclusion

This cross-sectional survey study sheds light on the readiness of community-dwelling older adults aged 75 years or older in South Tyrol, Italy, to adopt digital health technologies. While older adults reported being open to embracing these technologies, readiness to adopt telemedicine or wearables was higher than readiness to adopt smartphones with texting apps or assistant robots. Readiness to adopt digital health technologies among older adults was explained by sociodemographic, health-related, and lifestyle factors, with higher age consistently associated with less willingness to adopt digital health technologies, particularly among individuals aged 85 years and older. The use of computers, smartphones, and the internet in everyday life appears to be the most significant driver of readiness to adopt digital health technologies. Key stakeholders, including health care providers, technology developers, and policymakers, require a nuanced understanding of the readiness of older adults for digital health technologies. Among older people, tailored approaches addressing the needs of diverse demographic segments could further enhance the adoption rates. Collaborative initiatives, blending technological innovation with elder-friendly interfaces, and protective policies are vital for the seamless integration of digital health technologies into the lives of older people. Further, longitudinal studies are needed to explore how attitudes toward and the use of digital health technologies evolve among older adults over time. Qualitative research is necessary to gain a deeper understanding of the differential adoption readiness of older adults, including their language differences.

## Appendix

Multimedia Appendix 1 Supplementary tables.

## Abbreviations

ASTAT: The Provincial Institute of Statistics (Istituto Provinciale di Statistica – Landesinstitut für Statistik)
OR: odds ratio
PRISMA-7: Program of Research on Integration of Services for the Maintenance of Autonomy

## Data Availability

The data sets generated and analyzed during this study are available from the corresponding author upon reasonable request.
