# Supplementary material for: Community-Dwelling Older Adults’ Readiness for Adopting Digital Health Technologies: Cross-Sectional Survey Study
Source: JMIR Form Res. 2024 Apr 30;8:e54120. doi: 10.2196/54120 (PMC11094597; doi:10.2196/54120)
Supplement: Multimedia Appendix 1 [file formative_v8i1e54120_app1.pdf]

Supplementary Table. Differences between older people's readiness to adopt digital health technologies (N=1,521)

	Telemedicine		*P* [a]	Smartphone and texting applications		*P* [a]	Wearables		*P* [a]	Assistant robot		*P* [a]
	Yes or Maybe	No		Yes or Maybe	No		Yes or Maybe	No		Yes or Maybe	No	
**Gender** - % (n)			<0.001			<0.001			0.70			0.29
Female	51.7 (370)	48.3 (345)		46.4 (343)	54.6 (361)		68.9 (396)	31.1 (343)		42.3 (396)	57.7 (343)	
Male	59.8 (482)	40.2 (324)		56.3 (466)	43.7 (396)		69.9 (361)	30.1 (466)		45.1 (361)	54.9 (466)	
**Age** - % (n)			<0.001			<0.001			0.002			<0.001
75-84 years	63.1 (574)	36.9 (336)		59.7 (560)	40.3 (378)		72.4 (697)	27.6 (266)		47.9 (453)	52.1 (493)	
≥ 85 years	45.5 (278)	54.5 (333)		39.6 (249)	60.4 (379)		65.0 (415)	35.0 (239)		37.6 (238)	62.4 (395)	
**Native tongue**- % (n)			<0.001			<0.001			0.55			0.01
German	50.5 (410)	49.5 (402)		46.8 (388)	53.2 (441)		68.9 (585)	31.1 (264)		44.2 (371)	55.8 (468)	
Italian	62.9 (404)	37.1 (238)		58.2 (390)	41.8 (280)		70.5 (477)	29.5 (200)		42.4 (282)	57.6 (383)	
Ladin	51.8 (29)	48.2 (27)		39.3 (22)	60.7 (34)		64.1 (41)	23 (35.9)		43.8 (28)	56.2 (36)	
Other	81.8 (9)	18.2 (2)		81.8 (9)	18.2 (2)		81.8 (9)	18.2 (2)		90.9 (10)	9.1 (1)	
**Citizenship** - % (n)			0.24			0.33			1.00			0.14
Italian	55.8 (839)	44.2 (664)		51.5 (796)	48.5 (750)		69.4 (1098)	30.6 (483)		43.5 (678)	56.5 (880)	
Other	72.2 (13)	27.8 (5)		65.0 (13)	35.0 (7)		70.0 (14)	30.0 (6)		61.9 (13)	38.1 (8)	
**Community** - % (n)			<0.001			<0.001			0.34			0.88
Rural	49.9 (346)	50.1 (347)		45.5 (323)	54.5 (387)		68.2 (495)	31.8 (231)		44.0 (317)	56.0 (403)	
Urban	61.1 (506)	38.9 (322)		56.8 (486)	43.2 (370)		70.5 (617)	29.5 (258)		43.5 (374)	485 (56.5)	
**Living situation** - % (n)			0.05			0.03			0.01			0.03

Living alone	52.2 (240)	47.8 (220)		47.3 (222)	52.7 (247)		65.1 (801)	34.9 (322)		39.5 (187)	60.5 (286)	
Living with partner/family	57.7 (612)	42.3 (449)		53.5 (587)	46.5 (510)		71.3 (311)	28.7 (167)		45.6 (504)	54.4 (602)	
**Children** - % (n)			0.36			0.13			0.52			0.13
Yes	55.4 (668)	44.6 (538)		50.6 (629)	49.4 (613)		69.9 (888)	30.1 (383)		44.8 (563)	55.2 (695)	
No	58.4(184)	41.6 (131)		55.6 (180)	44.4 (144)		67.9 (224)	32.1 (106)		39.9 (128)	193 (60.1)	
**Educational level** - % (n)			<0.001			<0.001			0.005			0.05
Below Highschool	50.8 (598)	49.2 (580)		46.5 (562)	53.5 (647)		67.7 (840)	32.3 (401)		42.4 (516)	701 (57.6)	
Highschool or higher	74.1 (254)	25.9 (89)		69.2 (247)	30.0 (110)		75.6 (272)	24.4 (88)		48.3 (175)	187 (51.7)	
**Financial resources** - % (n)			<0.001			<0.001			0.02			0.18
Excellent or good	63.0 (272)	37.0 (160)		57.3 (256)	42.7 (191)		71.2 (324)	28.8 (131)		47.2 (214)	52.8 (239)	
Adequate	57.0 (430)	43.0 (324)		53.0 (411)	47.0 (365)		71.2 (562)	28.8 (227)		42.9 (333)	57.1 (444)	
Insufficient or low	44.8 (150)	55.2 (185)		41.4 (142)	58.6 (201)		63.3 (226)	36.7 (131)		41.3 (144)	205 (58.7)	
**Overall optimism** - % (n)			0.02			0.01			0.92			0.47
Yes	57.3 (743)	42.7 (554)		53.0 (707)	47.0 (626)		69.5 (945)	30.5 (414)		44.2 (592)	55.8 (748)	
No	48.7 (109)	51.3 (115)		43.8 (102)	56.2 (131)		69.0 (167)	31.0 (75)		41.4 (99)	58.6 (140)	
**Health status** - % (n)			0.002			<0.001			0.92			0.22
Poor or moderate	52.8 (480)	47.2 (429)		47.9 (361)	51.1 (269)		69.6 (664)	30.4 (290)		42.4 (399)	57.6 (541)	
Good or very good	60.8 (372)	39.2 (240)		57.3 (361)	42.7 (269)		69.2 (448)	30.8 (199)		45.7 (292)	54.3 (347)	
**Frailty (PRISMA-7)** - % (n)			<0.001			<0.001			0.70			0.17
Yes	47.9 (239)	52.1 (260)		41.2 (213)	58.8 (304)		68.7 (365)	31.3 (166)		41.3 (215)	58.7 (306)	

No	60.0 (613)	40.0 (409)		56.8 (596)	43.2 (453)		69.8 (747)	30.2 (323)		45.0 (476)	55.0 (582)	
**Physical activity**			<0.001			<0.001			<0.001			0.02
2 hours or more a week	64.4 (432)	35.6 (239)		61.1 (422)	38.9 (269)		73.7 (521)	26.3 (186)		47.4 (327)	52.6 (363)	
Less than 2 hours a week	50.9 (308)	49.1 (297)		47.7 (297)	52.3 (325)		69.3 (440)	30.7 (195)		42.1 (265)	57.9 (364)	
Never	45.7 (112)	54.3 (133)		35.6 (90)	64.4 (163)		58.3 (151)	41.7 (108)		38.1 (99)	61.9 (161)	
**Home care assistance (e.g., from family, nursing team and/or private family assistant)**			<0.001			<0.001			0.25			0.33
Yes	59.7 (543)	40.3 (366)		57.0 (532)	401 (43.0)		68.3 (656)	31.7 (304)		42.7 (402)	57.3 (539)	
No	50.5 (309)	49.5 (303)		43.8 (277)	356 (56.2)		71.1 (456)	28.9 (185)		45.3 (289)	54.7 (349)	
**Using computer, tablet**			<0.001			<0.001			<0.001			<0.001
Yes	80.6 (383)	19.4 (92)		77.5 (382)	111 (22.5)		77.7 (393)	22.3 (113)		52.8 (261)	47.2 (233)	
No	44.8 (469)	55.2 (577)		39.8 (427)	60.2 (646)		65.7 (719)	34.3 (376)		39.6 (430)	60.4 (655)	
**Using smartphone**			<0.001			<0.001			<0.001			<0.001
Yes	76.1 (467)	23.9 (147)		73.0 (467)	173 (27.0)		76.3 (502)	23.7 (156)		51.7 (333)	48.3 (311)	
No	42.4 (385)	57.6 (522)		36.9 (342)	584 (63.1)		64.7 (610)	35.3 (333)		38.3 (358)	61.7 (577)	
**Using internet**			<0.001			<0.001			<0.001			<0.001
Yes	79.5 (473)	20.5(122)		77.1 (476)	141 (22.9)		77.0 (488)	23.0 (146)		52.8 (326)	47.2 (292)	
No	40.9 (379)	59.1 (547)		35.1 (333)	64.9 (616)		64.5 (624)	35.5 (343)		38.0 (365)	62.0 (596)	

[a] Fisher's exact-Test
